# Efficacy of moderately hypofractionated simultaneous integrated boost intensity-modulated radiotherapy combined with temozolomide for the postoperative treatment of glioblastoma multiforme: a single-institution experience

**DOI:** 10.1186/s13014-019-1305-1

**Published:** 2019-06-13

**Authors:** Liangzhi Zhong, Lu Chen, Shengqing Lv, Qingrui Li, Guangpeng Chen, Wen Luo, Pu Zhou, Guanghui Li

**Affiliations:** 1 Cancer Research Institute of the Chinese People’s Liberation Army, Xinqiao Hospital, Army Medical University, Chongqing, 400037 China; 2 Department of neurosurgery, Xinqiao Hospital, Army Medical University, Chongqing, 400037 China; 3 Biobank, Southwest Hospital, Army Medical University, Chongqing, 400038 China

**Keywords:** Glioblastoma multiforme, Simultaneous integrated boost intensity-modulated radiotherapy, Temozolomide, Toxicity, Efficacy

## Abstract

**Purpose:**

Despite recent advances in multimodal treatments, the prognosis of patients with glioblastoma multiforme (GBM) remains poor. The aim of this study was to evaluate the efficacy of moderately hypofractionated simultaneous integrated boost intensity-modulated radiotherapy (SIB-IMRT) combined with temozolomide (TMZ) for the postoperative treatment of GBM.

**Materials and methods:**

From February 2012 to February 2018, 80 patients with newly diagnosed and histologically confirmed GBM in our institute were reviewed retrospectively. All patients underwent complete resection or partial resection surgery and then received hypofractionated SIB-IMRT with concomitant TMZ followed by adjuvant TMZ. A total dose of 64 Gy over 27 fractions was delivered to the gross tumor volume (GTV), clinical target volume 1 (CTV1) received 60 Gy over 27 fractions, and CTV2 received 54 Gy over 27 fractions. The progression-free survival (PFS) and overall survival (OS) rates and the toxicities were evaluated. Prognostic factors were analyzed using univariate and multivariate Cox models.

**Results:**

The median follow-up was 16 months (range, 5~72 months). The median PFS was 15 months, and the 1-, 2-, and 3-year PFS rates were 56.0, 27.6, and 19.5%, respectively. The median OS was 21 months, and the 1-, 2-, 3-, and 5-year OS rates were 77.6, 41.6, 32.8, and 13.4%, respectively. The toxicities were mild and acceptable. Age, KPS scores and the total number of TMZ cycles were significant factors influencing patient survival.

**Conclusion:**

Moderately hypofractionated SIB-IMRT combined with TMZ is a feasible and safe treatment option with mild toxicity and good PFS and OS.

## Background

Glioblastoma multiforme (GBM) is the most common and aggressive malignant brain tumor in adults. The current standard of care for newly diagnosed GBM is maximal surgical resection followed by radiotherapy (RT) in association with concomitant and adjuvant temozolomide (TMZ); currently; the median OS is 14.6 months [1]. Despite advances in microsurgical techniques, RT and chemotherapy, prognosis remains poor, and more than 90% of patients die within 5 years. The pattern of failure is primarily tumor recurrence. Studies have shown that approximately 90% of recurrence occurs within the original treatment field [2–5], which could result from insufficient therapeutic doses [6]. Therefore, high-dose radiation seems reasonable to improve local control with the hope of improving survival.

Dose escalation can be achieved by increasing the total dose or the dose per fraction. Increasing the total dose seems logical, but a previous study suggested that high-dose conformal RT did not improve survival despite the total dose delivered being up to 90 Gy [7]. Hypofractionation is an alternative method to increase the biological effect of RT, which can achieve increased cell kill from a higher dose per fraction and offset the acceleration of tumor cell repopulation by shortening the overall treatment time [8]. However, the larger the dose per fraction delivered, the higher the rate of radionecrosis is [9, 10], which may hinder the application of hypofractionated RT. To reduce the maximum dose to the organs at risk and increase the maximum dose to the target volumes, the simultaneous integrated boost intensity-modulated radiotherapy (SIB-IMRT) technique was developed [11].

Previous studies have investigated the efficacy and safety of hypofractionated SIB-IMRT combined with TMZ [12–14]. In 2012, we began to use moderately hypofractionated SIB-IMRT combined with concomitant and adjuvant TMZ in postoperative patients with GBM. The aim of the current study is to report on the efficacy of this regimen, including treatment-related toxicity, local recurrence, progression-free survival (PFS), and overall survival (OS).

## Materials and methods

### Patient population

From February 2012 to February 2018, a total of 526 patients were diagnosed with glioma in our hospital. Among these patients, 207 were diagnosed with high-grade glioma. Patients who had histologically confirmed GBM (World Health Organization [WHO] grade IV astrocytoma) were included in this study. Patients with GBM had undergone surgical resection before RT in our institution. Therefore, this study included only postoperative patients. No limitations were placed on Karnofsky performance status (KPS), age, lesion location or extent of surgery. This was a retrospective analysis, and approval was obtained from the institutional review board and ethics committee.

### Treatment planning and delivery

RT was initiated as soon as possible after surgery, within 6 weeks in most cases. Patients were placed in supine position with a customized immobilization device. Each patient underwent a spiral computed tomography (CT) scan with 3 mm slice thickness. The CT images were then transferred to and registered in the treatment planning system (Varian Medical System, Palo Alto, USA). The gross tumor volume (GTV) was defined as all contrast-enhancing lesions on the postoperative MRI T1-weighted images and the postoperative cavity with the latter fused with computed tomography images for treatment planning. The clinical target volume (CTV) 1 was defined as GTV plus a 1~2-cm margin, including surrounding edema on T2-weighted fluid-attenuated inversion recovery MRI. If the edema was beyond 2 cm from the GTV, the margin was expanded to include all of the area exhibiting edema. CTV2 was defined as CTV1 plus a 1~2-cm margin. The planning target volume (PTV), including PGTV, PCTV1, and PCTV2, was defined as the respective above target volume plus a 0.3-cm margin. The margin could be modified to a smaller margin if there were organs at risk (OARs), such as the brain stem, optical pathway, or spinal cord, or if there were anatomical barriers, such as the dura, tentorium, and falx cerebri.

**Fig. 1:**
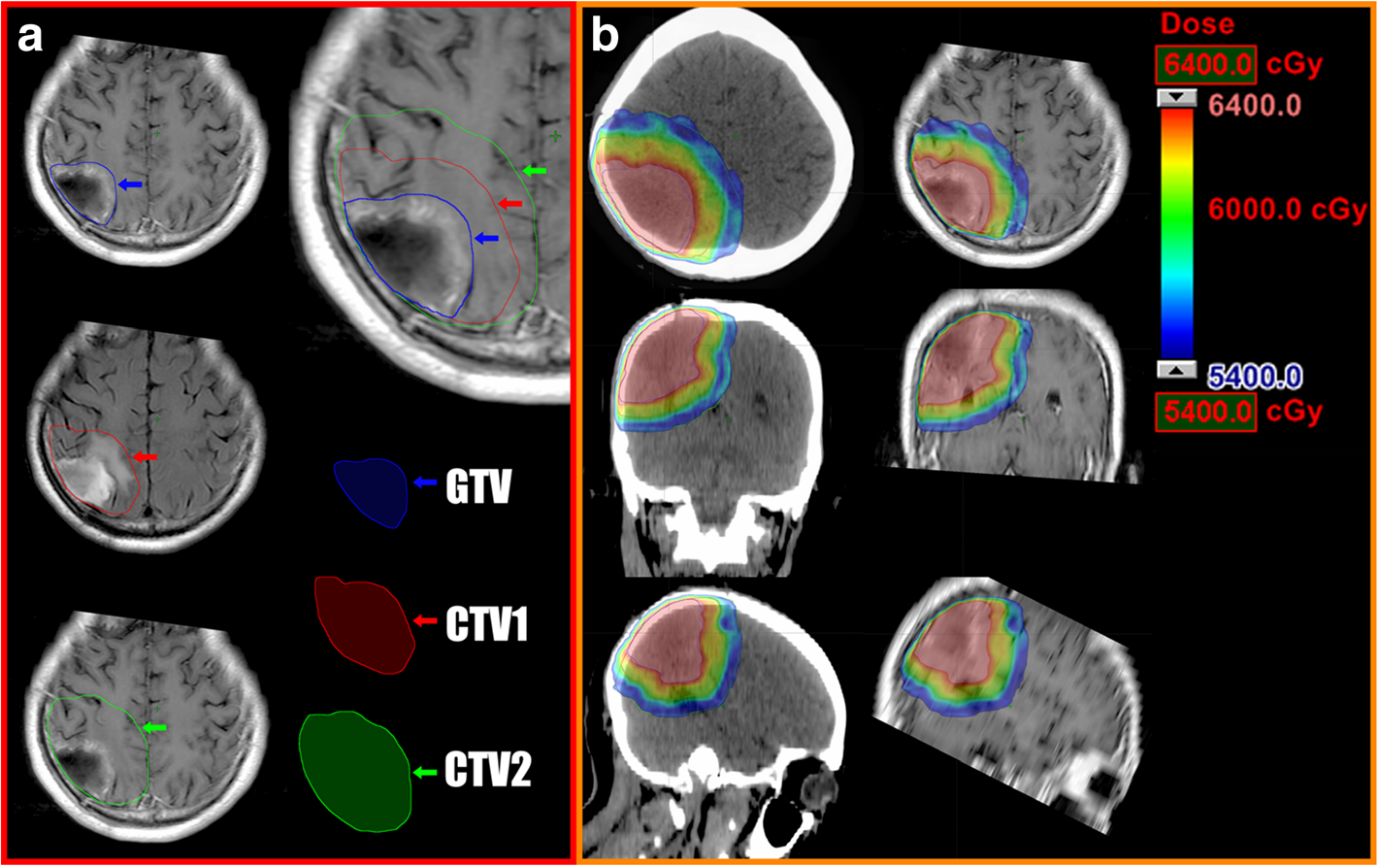
The target volume delineation and isodose distribution of a representative patient who received hypofractionated SIB-IMRT. **a** GTV is identified with a blue arrow, including the contrast-enhanced lesion on the postoperative T1-weighted MR images and the postoperative cavity; CTV1 is identified with a red arrow, including GTV plus a 1-cm margin and the surrounding edema on T2-weighted fluid-attenuated inversion recovery MRI; CTV2 is identified with a blue arrow, including CTV1 plus a 2-cm margin. **b** Isodose distribution in CT and MRI, showing the target volumes receiving 64 Gy (red), 60 Gy (green), or 54 Gy (blue)

Treatment using a step-and-shoot technique with 6 MV photon beams was delivered with an MLC-equipped megavoltage linear accelerator (Trilogy Linac, Varian, USA). Daily doses of 2.4 Gy, 2.2 Gy, and 2.0 Gy were delivered to PGTV, PCTV1, and PCTV2 with a total dose of 64 Gy, 60 Gy and 54 Gy, respectively, in 27 fractions over 6 weeks. The planning goal was the prescription dose encompassing at least 95% of the PTV, and no more than 10% of the PTV received more than 110% of the prescribed dose. The critical structures included the brain stem, optic chiasm, lens, optic nerves, and hippocampus. The upper dose constraints for the brain stem, optic nerves and chiasm are 54 Gy, the maximum for lenses is 5 Gy, the maximum dose for hippocampus is less than 24 Gy, and the mean dose was less than 12 Gy. The targets and dose distribution in a representative patient are shown in Fig. 1.

All patients except for 5 patients, due to personal reasons, received concomitant TMZ (75 mg/m^2^/day) every day during RT. After a 4-week break, the patients received adjuvant TMZ (150–200 mg/m^2^/day) for 5 days every 28 days. The total number of TMZ cycles were determined by oncologists according to the patients’ general condition, compliance, economic situation and disease progression.

### Follow-up

Patients were followed weekly during the treatment period, with a medical history, physical examination, and complete blood test, and then patients were followed every month after finishing the treatment for at least 3 months. All patients were followed routinely with neurological examinations and MRI imaging at 3- to 6-month intervals after the treatment. MR spectroscopy and MR perfusion of the brain were not routinely used, except when in doubt about tumor progression or necrosis. Acute toxicities were scored using the Common Terminology Criteria for Adverse Events, version 4.0. Late toxicities were scored according to the RTOG/EORTC toxicity criteria.

Tumor progression was defined as new lesions showing enhancement outside of the radiation field within the first 12 weeks of RT completion, a 25% increase in the size of one or more measurable lesions or the appearance of new lesions more than 12 weeks after RT completion [15]. Distant metastasis was defined as new lesions that occurred outside the brain. The follow-up duration was defined as the time from the date of surgery to the last date of follow-up for surviving patients or to the date of death. The last follow-up date was August 1, 2018.

### Statistical analysis

The Kaplan-Meier method was used to evaluate the rates of local recurrence, PFS and OS. The log-rank test and Cox regression method were used for univariate and multivariate analyses, respectively. Statistical analysis was performed with SPSS software (version 20.0; SPSS Inc., Chicago, IL), and *P* < 0.05 was considered statistically significant.

## Results

Between February 2012 and February 2018, 80 patients were included in the analysis. The patients’ characteristics are listed in Table 1. Fifty patients were men, and 30 patients were women. The median age was 50 years (range, 24~75 years). Forty-four patients received partial resection surgery, and 36 patients received complete resection surgery. Among these patients, 75 were treated with hypofractionated SIB-IMRT and concomitant TMZ, while 5 patients were not treated with concomitant TMZ for personal reasons. Sixty-four patients received adjuvant TMZ, while 16 patients did not receive adjuvant TMZ for economic reasons. The median number of adjuvant chemotherapy cycles was 5 (range, 0~12).

**Table 1:** Demographic and Baseline Clinical Characteristics of Patients

Variables	Patients N. (*N* = 80)
Sex	
Male	50 (62.5%)
Female	30 (37.5%)
Age	
Median (range)	50 (24–75)
< 50	39 (48.8%)
≥ 50	41 (51.2%)
Extant of surgery	
Partial resection	44 (55.0%)
Complete resection	36 (45.0%)
Concurrent TMZ	
Yes	75 (93.8%)
No	5 (6.2%)
KPS scores	
90–100	41 (51.2%)
80	16 (20.0%)
≤ 70	23 (28.8%)
TMZ cycles	
Median (range)	5 (0–12)
< 6	42 (52.5%)
≥ 6	38 (47.5%)

### Efficacy

**Fig. 2:**
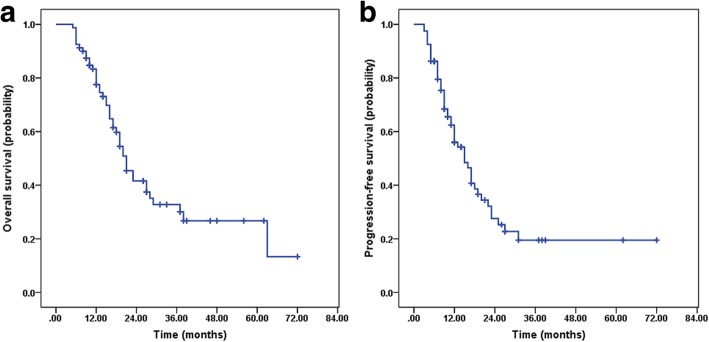
Kaplan-Meier estimates. **a** Progression-free survival (PFS). **b** Overall survival (OS)

**Fig. 3:**
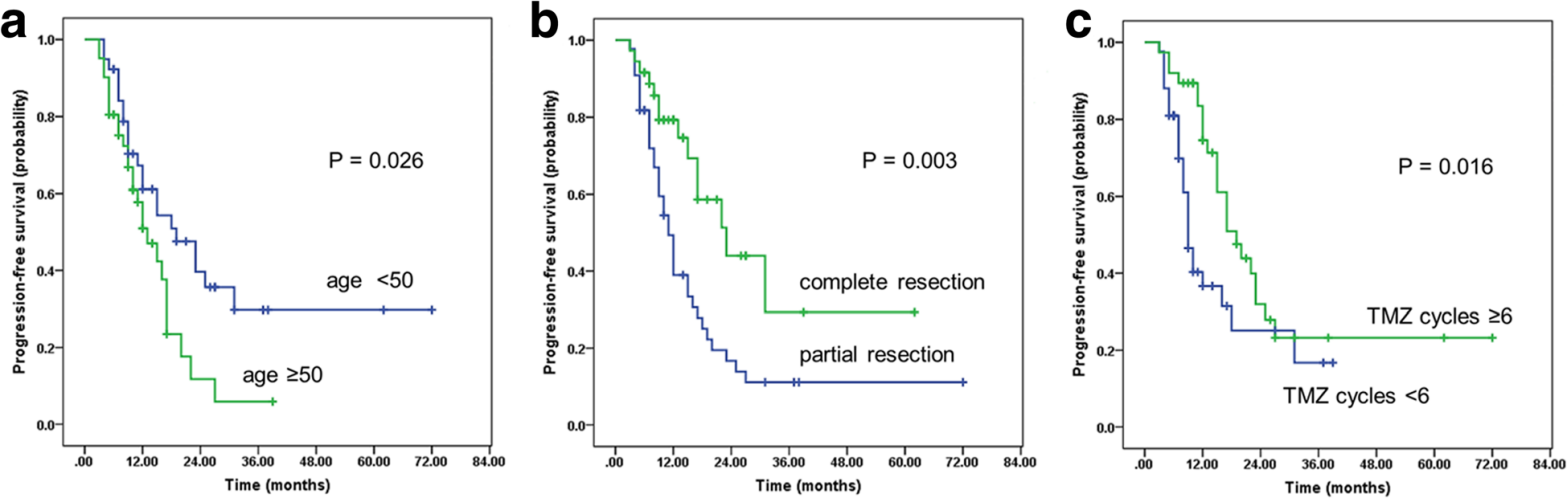
Kaplan-Meier estimates of progression-free survival (PFS). **a** PFS for patients of different ages. **b** PFS for patients who received different extents of surgical resection. **c** PFS for patients with different numbers of adjuvant TMZ cycles

**Fig. 4:**
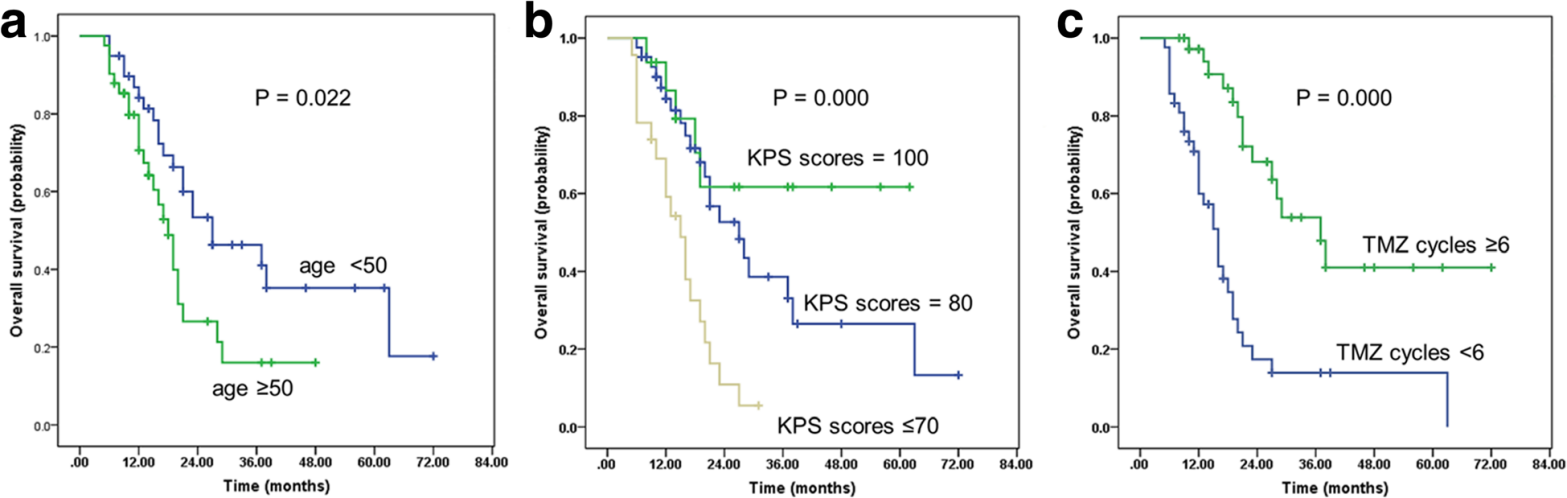
Kaplan-Meier estimates of overall survival (OS). **a** OS for patients of different ages; **b** OS for patients with different KPS scores; **c** OS for patients with different numbers of adjuvant TMZ cycles

After a median follow-up of 16 months (range, 5~72 months), 45 (56.3%) patients had died, and 49 (64.8%) patients exhibited tumor progression. The median OS and PFS rates were 21 months (95% confidence interval [CI], 17.5–24.4) and 15 months (95% CI, 11.0–18.9), respectively. The 1-, 2-, and 3-year rates of PFS among the whole group were 56.0, 27.6, and 19.5%, respectively (Fig. 2a). The 1-, 2-, 3-, and 5-year rates of OS were 77.6, 41.6, 32.8, and 13.4%, respectively (Fig. 2b). Age, extent of surgery, KPS scores and the total number of TMZ cycles were significant factors influencing PFS and OS in the univariate analyses (Table 2). In the multivariate analysis, age, extent of surgical resection and the total number of TMZ cycles were significant factors influencing PFS (Fig. 3); age, KPS scores and the total number of TMZ cycles were significant factors influencing OS (Fig. 4).

**Table 2:** Univariate and multivariate analysis of PFS and OS in all patients

Variable	Univariate		Multivariate					
	PFS	0S	PFS			OS		
	P	P	HR	95%CI	P	HR	95%CI	P
Sex	0.329	0.429						
Male								
Female								
Age	0.026^a^	0.022^a^						
< 50			RL			RL		
≥ 50			1.894	(1.049,3.419)	0.034	1.999	(1.072,3.727)	0.029
Extent of surgery	0.003^a^	0.040^a^						
PR			RL			RL		
CR			0.370	(0.198,0.692)	0.002			
KPS scores	0.036^a^	0.000^a^						
90–100						RL		
80						0.273	(0.137,0.542)	0.000
≤ 70						0.265	(0.095,0.738)	0.011
Concomitant TMZ	0.945	0.113						
Yes								
No								
TMZ cycles	0.016^a^	0.000^a^						
< 6			RL			RL		
≥ 6			0.495	(0.275,0.890)	0.019	0.246	(0.124,0.488)	0.000

*Abbreviations*: *TMZ* temozolomide, *PR* partial resection, *CR* complete resection, *RL* referent level

^a^Significant in univariate analysis

### Patterns of failure

Tumor progression was detected in 49 (61.3%) patients, and progression was identified on MRI in 36 of them (Table 3). Sixteen patients had tumor progression within the GTV, 2 patients developed new lesions within CTV1, 1 patient developed new lesions within CTV2, 11 patients developed new lesions outside the radiation field and 6 patients had multicentric progression (both within and outside of the radiation field). The progression sites of 13 patients were unknown because MRI findings were unavailable. The remaining 22 patients were alive with no evidence of progression.

**Table 3:** Patterns of recurrence

Sites of recurrence	Number of patients
Within GTV	16
Within CTV1	2
Within CTV2	1
Outside the target volume	11
Multicentric recurrence	6
Unknown	13

### Toxicity

The most common acute toxicities were nausea, fatigue, headache and hematologic toxicities, which were mainly in grade 1 or 2 and occurred during the concomitant RT and TMZ period. Five patients experienced grade 3 toxicity, including neutropenia (1 patient, 1.3%), anemia (2 patients, 2.5%), and thrombocytopenia (2 patients, 2.5%). No patients experienced grade 4 toxicity. The most common late adverse effects were cognitive disturbances, which occurred in 4 (5.0%) patients. Three patients (3.7%) developed radionecrosis. Two patients (2.5%) presented with progressive headache and dizziness 1 year after RT, and MRI showed increased enhancement. These patients underwent reoperation. The postoperative pathological examination showed only necrotic tissue and no presence of tumor tissue. One patient (1.2%) was symptom-free, but MRI showed increased enhancement 3 months after treatment; however, there was a lack of evidence of progression upon MR perfusion and MR spectroscopy, so a diagnosis of clinical radionecrosis was made. All patients completed the planned RT treatment. No treatment-related death occurred.

## Discussion

Previous studies have shown that hypofractionated SIB-IMRT combined with TMZ for the treatment of GBM has favorable outcomes on survival [12–14]. However, the larger the dose per fraction delivered, the higher the rate of cerebral necrosis is [10, 16]. Cho et al. reported that a slightly hypofractionated regimen in association with TMZ chemotherapy had encouraging survival rates without increasing the risk of unacceptable toxicity [12]. In this study, we showed that moderately hypofractionated SIB-IMRT combined with TMZ for the postoperative treatment of GBM had a good outcome, with a median OS of 21 months and a median PFS of 15 months. Sultanem et al. [17] conducted a study to evaluate the efficacy of hypofractionated SIB-IMRT in the treatment of 25 patients with GBM. A total of 60 Gy over 20 daily fractions of 3 Gy each were applied to the GTV, and the PTV received a minimum of 40 Gy over 20 fractions of 2 Gy each at its periphery. The median OS was 9.5 months, and the median PFS was 5.2 months. Acute toxicity occurred in 2 (8%) patients, and no patient developed late toxicity. Panet-Raymond et al. [13] carried out a study to observe the efficacy of SIB-IMRT combined with TMZ in 35 patients with GBM. During a 4-week period, doses of 60 Gy and 40 Gy were delivered in 20 fractions prescribed to the GTV and PTV, respectively. The median survival was 14.4 months, and the median disease-free survival was 7.7 months. The most common acute toxicity was moderate fatigue. No patient developed late toxicity. Mallick et al. [18] compared conventionally fractionated radiation therapy (CRT) and hypofractionated accelerated radiation therapy (HART) in 83 patients with GBM who were between the ages of 16 and 65 years old. The prescribed dose was 50 Gy over 25 fractions to CTV50, followed by a boost of 10 Gy over 5 fractions to CTV60 in the CRT arm. The prescribed dose was 60 Gy over 20 fractions to CTV60 and 50 Gy over 20 fractions to CTV50 in the HART arm. The median OS for the entire cohort was 23.4 months, but the median OS was not significantly different between the two arms, with a median OS of 18.07 and 25.18 months (*p* = 0.3). The median PFS for the entire cohort was 13.5 months. One patient (1.2%) had documented radionecrosis. Scoccianti et al. [19] evaluated the efficacy and toxicity of hypofractionated SIB combined with TMZ in 24 patients with relatively a good prognosis (recursive partitioning analysis classes III and IV). A total dose of 52.5 Gy over 15 fractions and 67.5 Gy over 15 fractions was delivered to the SIB volume. The median OS was 15.1 months, and the median PFS was 8.6 months. One patient (4.2%) developed radionecrosis of the brain parenchyma. Although our survival outcome seems to be superior to the results reported by most other studies, it is hard to compare our results with these studies directly because the definition of target volumes and fractionation schedules employed in among studies vary widely. In addition, other studies not only included patients who had received surgical resection but also those who received only biopsy, whereas our study included only patients who had received surgery. Only a well-designed randomized trial can confirm whether our regimen is comparable or superior to the standard treatment regimen.

Age, KPS scores, extent of surgical resection, and the addition of adjuvant chemotherapy have been shown to be significant prognostic factors [20–22]. Our results are similar to those previously reported. Age, KPS scores and the total number of adjuvant TMZ cycles were significant factors influencing OS in the univariate and multivariate analyses. The pairwise comparisons of OS within different KPS scores demonstrated that patients who had a higher KPS score exhibited improved survival. This result agreed with Scoccianti’s multicenter study [22] in which 1059 eligible patients were enrolled, and they concluded that patients with higher postoperative KPS scores had better survival. Adjuvant chemotherapy is another important prognostic factor. In the present study, we found that patients who received adjuvant TMZ for more than 6 cycles had a median survival of 28 months (95% CI 20.3–35.7, *p* = 0.004) compared to 16 months (95% CI 13.5–18.5, *p* = 0.004) in patients who received fewer than 6 cycles of adjuvant TMZ. This finding is consistent with that in Darlix’s study [23], which showed that prolonged TMZ administration may improve PFS and OS.

The major cause of failure in this study was tumor progression, and no patients had distant metastases. The most common site of recurrence was within the GTV. This finding is in concordance with that observed in other studies [24–26], which suggested that the majority of first GBM relapses following RT with TMZ occurred near the original contrast-enhanced mass. We found that the most common site of recurrence outside the target volume was the corpus callosum and the area close to the lateral ventricle, which occurred in 9 patients. Tumors can reportedly diffuse in white matter along the fiber bundles, such as the longitudinal fasciculi, uncinate fasciculus, visual bundles, corona radiata, etc. [27]. This phenomenon suggests that the current target volume in the area of fiber bundles may not be sufficient. To confirm our results, a randomized phase II trial (chiCTR1800014396) was conducted, and the prospective study will compare the moderately hypofractionated SIB-IMRT regimen with conventionally fractionated RT. The target volume will be delineated according to the patient’s neuroanatomy.

It has been reported previously that the acute and late toxicities of SIB-IMRT combined with TMZ in patients with GBM were mild [12–14, 17–19], as was the case in our study. The most common acute toxicities during the period of concomitant RT and TMZ were nausea, fatigue, headache and hematologic toxicities; only 5 patients (6.3%) experienced grade 3 toxicity, and no patients experienced grade 4 acute toxicities. Three patients (3.7%) developed radionecrosis. In addition, all patients finished the planned RT. No treatment-related deaths occurred. Therefore, the toxicity of the treatment modality used in our study was mild and tolerable.

The strengths of this study include the relatively large number of patients analyzed. In addition, we adopted moderately hypofractionated RT, which may improve the efficacy of RT while minimizing toxicity. However, the study has some limitations. First, this retrospective study relied heavily on medical records, and some bias exists in the course of data collection. Furthermore, previous studies have shown that O6-methylguanine-DNA-methyltransferase (MGMT) promoter methylation is a significant prognostic biomarker. Unfortunately, because molecular subtyping is not routinely performed in our institution, the MGMT status was not explored.

## Conclusions

Moderately hypofractionated SIB-IMRT combined with TMZ appears to be an effective and safe regimen for postoperative patients with GBM. This novel RT schedule may be an alternative treatment mode for GBM.

## Data Availability

The datasets used and analyzed during the current study are available from the corresponding author on reasonable request.

## Abbreviation

CTV: Clinical target volume
GBM: Glioblastoma multiforme
GTV: Gross tumor volume
KPS: Karnofsky performance status
MGMT: O6-methylguanine-DNA-methyltransferase
OARs: Organs at risk
OS: Overall survival
PFS: Progression-free survival
PTV: Planning target volume
RT: Radiotherapy
SIB-IMRT: Simultaneous integrated boost intensity-modulated radiotherapy
TMZ: Temozolomide
